# Supplementary material for: Of eyes and embryos: subfunctionalization of the CRX homeobox gene in mammalian evolution
Source: Proc Biol Sci. 2019 Jul 24;286(1907):20190830. doi: 10.1098/rspb.2019.0830 (PMC6661347; doi:10.1098/rspb.2019.0830)
Supplement: Supporting data figures [file rspb20190830supp2.pdf]

Electronic Supplementary Information 2

Royall AH, Frankenberg S, Pask AJ, Holland PWHH.

Of eyes and embryos: subfunctionalisation of the CRX homeobox gene in mammalian evolution.

Proceedings of the Royal Society B.
DOI 10.1098/rspb.2019.0830

(A) SRA identifiers for mouse preimplantation transcriptome datasets.

(B) Maximum-likelihood tree of CRX protein sequences from across metazoans shows divergence of sequence in eutherian mammals.

(C) Homeodomains of mouse ETCHbox genes do not show compensatory changes for the amino acids substituted in eutherian CRX homeodomain.

(D) RT-PCR for *CRX* expression in fat-tailed dunnart adult tissues.

(E) Immunocytochemistry staining for V5-tagged CRX proteins in MEFs shows nuclear localisation and demonstrates correct translation frame.

(F) TPM for ectopically expressed genes and chemical selection gene (puromycinR) shows there is no discrepancy in the levels of ectopic expression between samples, and that there is no endogenous expression of mouse *Crx* in the MEFs used.

(G) Mouse preimplantation embryo transcriptome profiles generated using MFuzz.

A

SRA identifiers for mouse preimplantation transcriptome datasets.

Species	Tissue	SRA study	Run ID
Mmus	Oocyte	SRP03454	SRR1051273
Mmus	Oocyte	SRP03454	SRR1051274
Mmus	Oocyte	SRP03454	SRR1051275
Mmus	Oocyte	SRP03454	SRR1051276
Mmus	Oocyte	SRP03454	SRR1051277
Mmus	Zygote	SRP03454	SRR1567937
Mmus	Zygote	SRP03454	SRR1051279
Mmus	Zygote	SRP03454	SRR1051278
Mmus	2Cell	SRP03454	SRR1051282
Mmus	2Cell	SRP03454	SRR1051281
Mmus	2Cell	SRP03454	SRR1051280
Mmus	4Cell	SRP03454	SRR1567921
Mmus	4Cell	SRP03454	SRR1567920
Mmus	4Cell	SRP03454	SRR1567919
Mmus	8Cell	SRP03454	SRR1567924
Mmus	8Cell	SRP03454	SRR1567923
Mmus	8Cell	SRP03454	SRR1567922
Mmus	Morula	SRP03454	SRR2048257
Mmus	Morula	SRP03454	SRR2048256
Mmus	Morula	SRP03454	SRR2048255
Mmus	Blastocyst	SRP03454	SRR1567925
Mmus	Blastocyst	SRP03454	SRR1567926
Mmus	Blastocyst	SRP03454	SRR1567927
Mmus	Blastocyst	SRP03454	SRR1567928
Mmus	E7.5	SRP03776	SRR1168503

B

Maximum-likelihood tree of CRX protein sequences from across metazoans shows divergence of sequence in eutherian mammals.

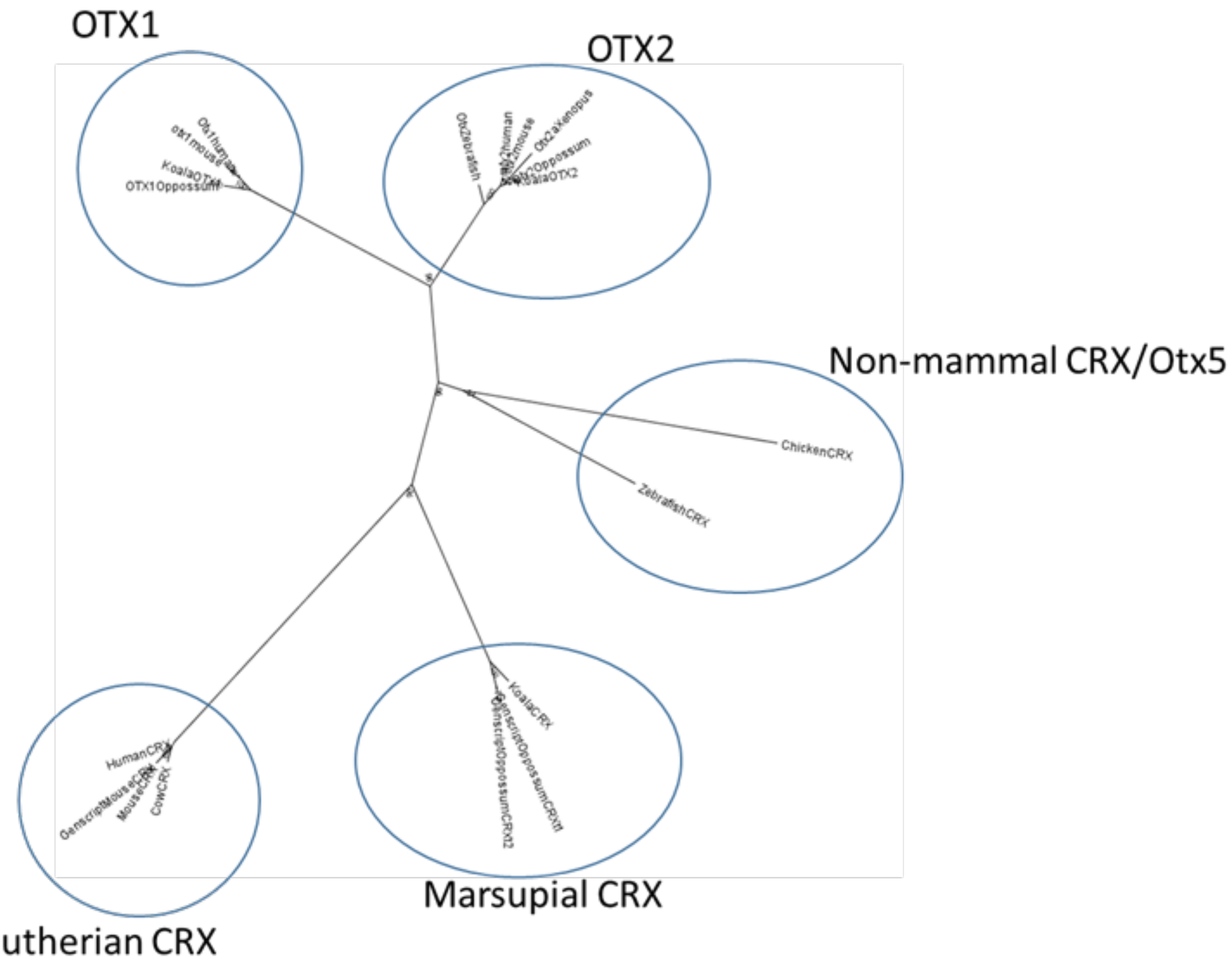

C

Homeodomains of mouse ETCHbox genes do not show compensatory changes for the amino acids which are lost in eutherian *CRX* homeodomain (black boxes). H = Human *CRX*, O = Opossum *CRX*.

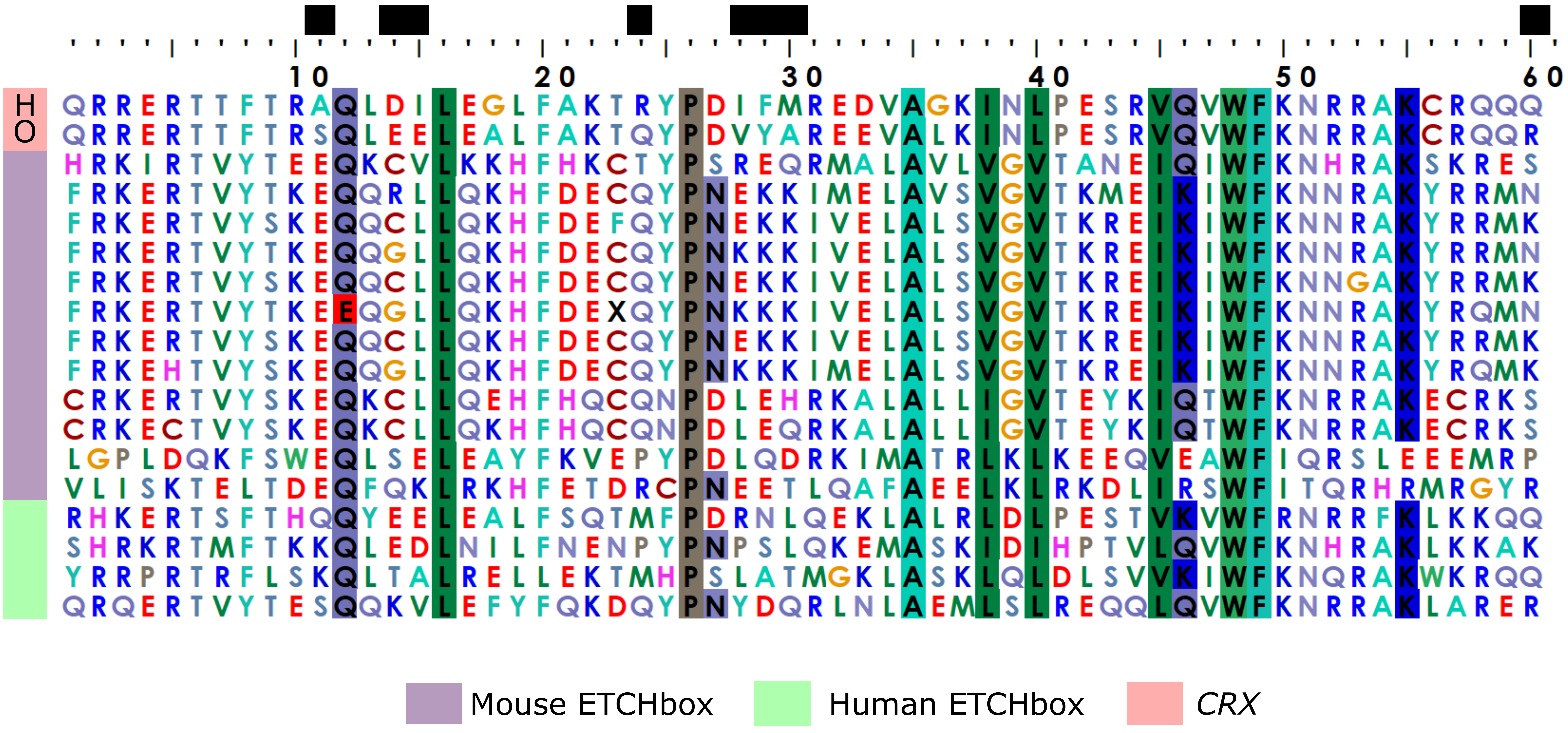

D

Reverse-Transcriptase PCR for *CRX*expression in fat-tailed dunnart adult tissues.

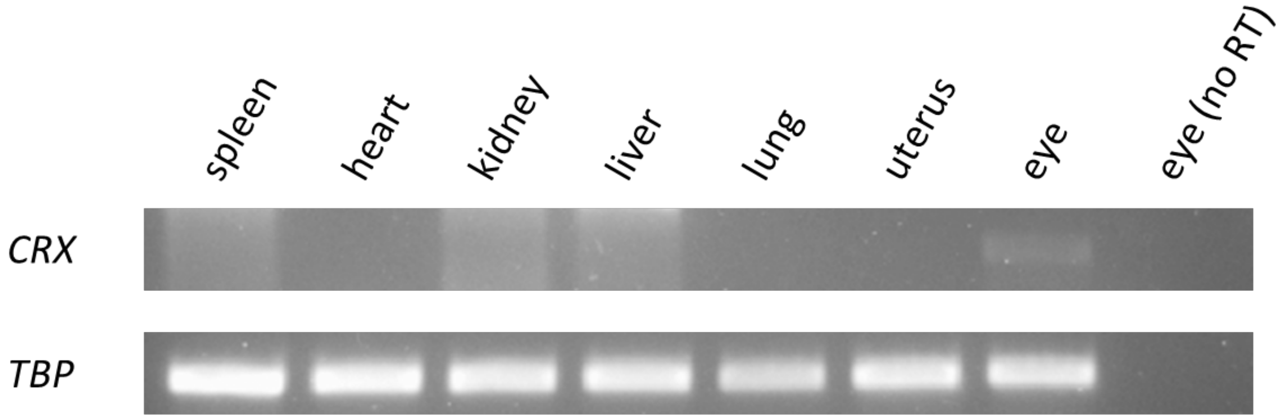

E

Metatherian and eutherian CRXectopic expression in MEFs is localised to the nucleus

The Opossum (*Monodelphis domestica*) is often used as a tool for investigating the evolution of mammals as it is thought to possess ancestral characteristics of the early metatherians. In order to study the role of *Crx* in regulating the transcriptome, and whether there is any distinction be tween the effect of metatherian and eutherian *Crx*, we ectopically expressed either Monodelphis domestica or human *Crx*genes in primary mouse cells and carried out Poly-A RNA-sequencing.

As homeodomain proteins such as *Crx* function as transcription factors, it is important to confirm that the ectopically expressed protein localises to the nucleus. To test this we ectopically expressed a both proteins of interest with a V5 tag in primary MEFs and visalised the cellular localisation of the fusion protein throu gh immunistaining against the tag. It is particularly important in this case to confirm the sub -cellular protein localisation following over -expression as we are studying genes in the cells of a foreign species meaning it cannot necessarily be assumed that modifications and mechanisms that confer nuclear localisation are transferable between species. The evident nuclear localisation of both proteins in a heterologous system implies that the assumed *in vivo* function may be recapitulated in our experimental system.

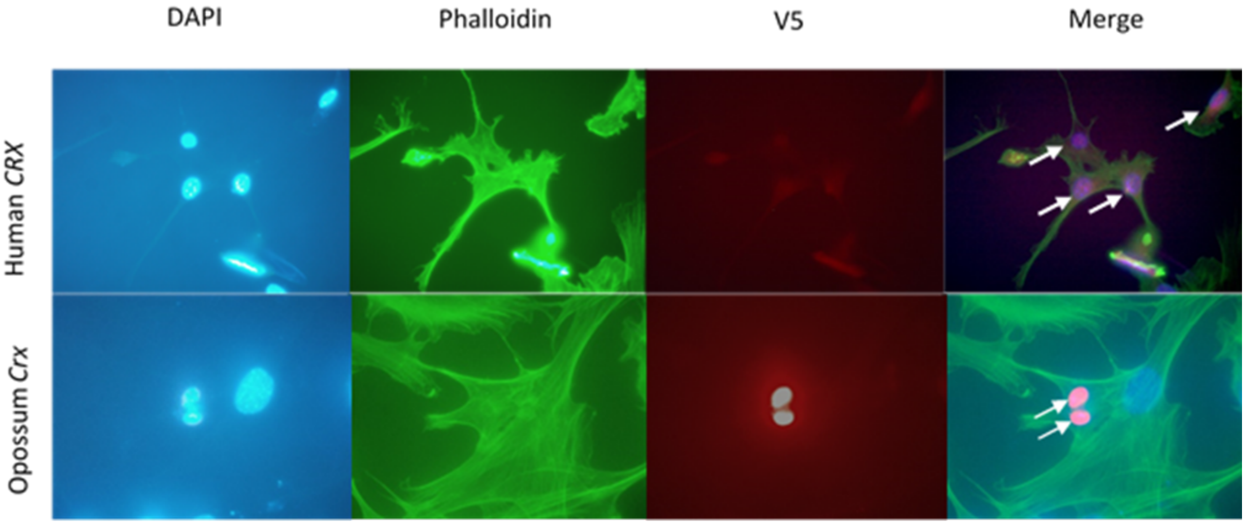

F

TPM for ectopically expressed genes and chemical selection gene (puromycinR) shows there is no discrepancy in the levels of ectopic expression between samples, and that there is no endogenous expression of mouse *Crx*in the MEFs used.

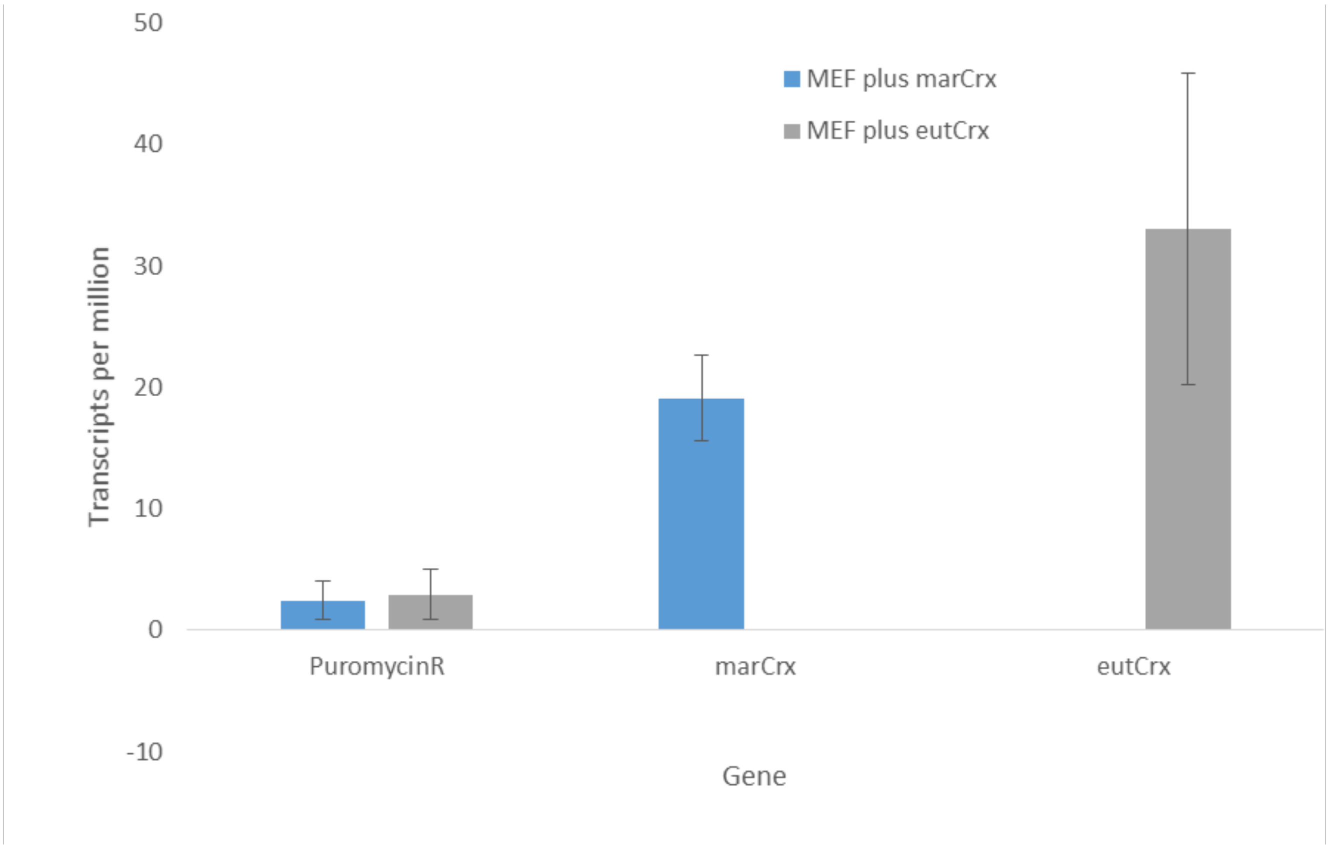

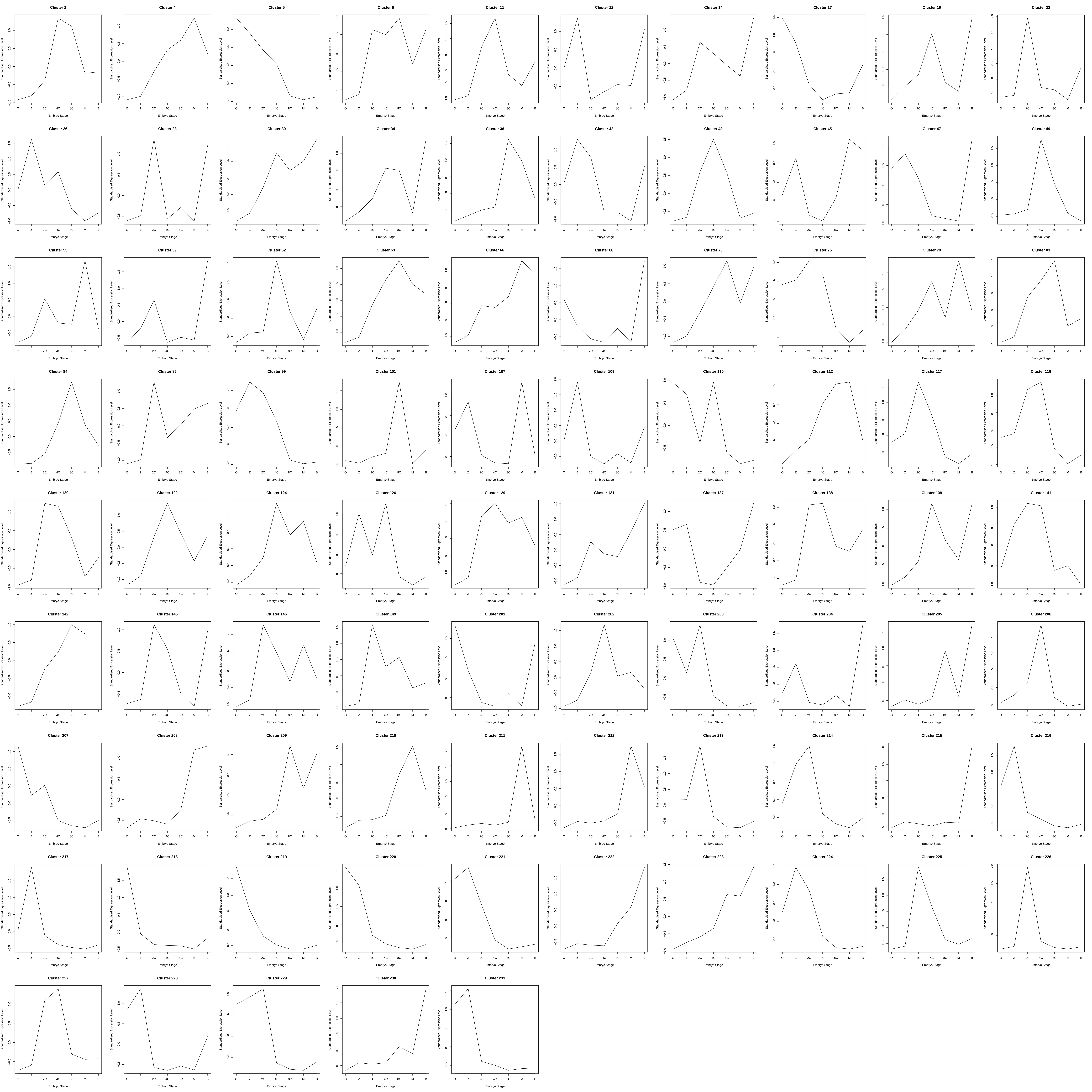

Cluster 2
Cluster 4
Cluster 5
Cluster 6
Cluster 11
Cluster 12
Cluster 14
Cluster 17
Cluster 19
Cluster 22
Cluster 26
Cluster 28
Cluster 30
Cluster 34
Cluster 36
Cluster 42
Cluster 43
Cluster 45
Cluster 47
Cluster 49
Cluster 53
Cluster 59
Cluster 62
Cluster 63
Cluster 66
Cluster 68
Cluster 73
Cluster 75
Cluster 79
Cluster 83
Cluster 84
Cluster 86
Cluster 99
Cluster 101
Cluster 107
Cluster 109
Cluster 110
Cluster 112
Cluster 117
Cluster 119
Cluster 120
Cluster 122
Cluster 124
Cluster 126
Cluster 129
Cluster 131
Cluster 137
Cluster 138
Cluster 139
Cluster 141
Cluster 142
Cluster 145
Cluster 146
Cluster 149
Cluster 201
Cluster 202
Cluster 203
Cluster 204
Cluster 205
Cluster 206
Cluster 207
Cluster 208
Cluster 209
Cluster 210
Cluster 211
Cluster 212
Cluster 213
Cluster 214
Cluster 215
Cluster 216
Cluster 217
Cluster 218
Cluster 219
Cluster 220
Cluster 221
Cluster 222
Cluster 223
Cluster 224
Cluster 225
Cluster 226
Cluster 227
Cluster 228
Cluster 229
Cluster 230
Cluster 231
Standardised Expression Level
Embryo Stage
O Z 2C 4C 8C M B
